# Comparative Efficacy of a Fungal Entomopathogen with a Broad Host Range against Two Human-Associated Pests

**DOI:** 10.3390/insects13090774

**Published:** 2022-08-26

**Authors:** Aaron R. Ashbrook, Aram Mikaelyan, Coby Schal

**Affiliations:** Department of Entomology and Plant Pathology, North Carolina State University, 100 Derieux Place, Raleigh, NC 27695, USA

**Keywords:** bed bugs, German cockroaches, *Beauveria bassiana*, entomopathogens, bioassays

## Abstract

**Simple Summary:**

Bed bugs and German cockroaches have adapted to thrive in human structures. In the present study, we use different techniques to expose bed bugs and German cockroaches to *Beauveria bassiana*, a fungal pathogen that only infects insects, to test their susceptibility to infection. The tests with bed bugs revealed that they were highly susceptible to fungal infections, no matter how we exposed them to the pathogen. The German cockroaches were only infected by fungi through certain routes of exposure. Fungal pathogens have the potential to control bed bugs but will require additional research and innovative technologies to be effective against cockroaches.

**Abstract:**

The ability of a fungal entomopathogen to infect an insect depends on a variety of factors, including strain, host, and environmental conditions. Similarly, an insect’s ability to prevent fungal infection is dependent on its biology, environment, and evolutionary history. Synanthropic pests have adapted to thrive in the indoor environment, yet they arose from divergent evolutionary lineages and occupy different feeding guilds. The hematophagous bed bug (*Cimex lectularius*) and omnivorous German cockroach (*Blattella germanica*) are highly successful indoors, but have evolved different physiological and behavioral adaptations to cope with the human-built environment, some of which also reduce the efficacy of fungal biopesticides. In order to gain greater insight into the host barriers that prevent or constrain fungal infection in bed bugs and German cockroaches, we tested different doses of *Beauveria bassiana* GHA through surface contact, topical application, feeding, and injection. Bed bugs were generally more susceptible to infection by *B*. *bassiana* with the mode of delivery having a significant impact on infectivity. The German cockroach was highly resilient to infection, requiring high doses of fungal conidia (>8.8 × 10^4^) delivered by injection into the hemocoel to cause mortality. Mortality occurred much faster in both insect species after exposure to surfaces dusted with dry conidia than surfaces treated with conidia suspended in water or oil. These findings highlight the importance of developing innovative delivery techniques to enhance fungal entomopathogens against bed bugs and cockroaches.

## 1. Introduction

Several times throughout their evolutionary history, fungi have evolved various strategies and adaptations to use living insects as resources [1]. Fungi in the order Entomophthorales, for example, are specialized to infect certain clades of insects and produce low amounts of conidia to cause subsequent infections [2]. Hypocrealean fungi such as *Metarhizium anisopliae* (Sorokin), *Beauveria bassiana* (Balsalmo), and *Isaria fumorosea* (Wize) have a wider host range than other fungi that infect insects and produce large quantities of conidia [1]. Some hypocrealean fungi can also become plant endophytes or saprotrophs, depending on resource availability [1,3], although they are primarily investigated because of their ability to cause epizootics within insect populations.

Potential insect hosts have evolved various physiological and behavioral adaptations to counter or prevent fungal infections. These adaptations can be linked to their mode of feeding and food source, habitat, and evolutionary history. For example, *Musca domestica* (Linnaeus) larvae, which live and feed in septic decaying materials, produce chitosan, which is anti-bacterial and can suppress the growth of several different fungi in vitro [4]. Phytophagous insects, such as the aphid *Aphis gossypii* (Glover), behaviorally avoid plants that are infected with entomopathogenic fungi [5]. Generalist predatory insects, such as the beetle *Coccinella septempunctata* (Linnaeus), avoid prey insects that are infected with *Beauveria bassiana* [6]. Similarly, parasitoids such as *Trybliographa rapae* (Westwood) avoid entomopathogen-infected hosts [7].

Indoor environments are harsh, and only a few species, considered pests, have adapted to exploit human-built niches [8]. Some of the challenges these species encounter are arid environments, pollution, local population bottlenecks and extinction [8], and habitat fragmentation that results in low genetic diversity within populations [9,10]. For example, populations of the hematophagous human-associated bed bug *Cimex lectularius* (Linnaeus) have significantly lower allelic diversity than that in bat-associated populations of the same species, suggesting an ancestral genetic bottleneck in the human-associated lineage [11]. The human-associated populations also have high frequencies of *kdr* mutations in response to exposure to DDT and pyrethroids [10,12], whereas the bat-associated populations do not [12]. Similarly, the omnivorous German cockroach *Blattella germanica* (Linnaeus) cannot fly, likely as an adaptation to the indoor environment, whereas its close relative *Blattella asahinai* (Mizukubo) lives outdoors and is capable of flight [13]. Surprisingly little is known about whether synanthropic species have evolved unique adaptations or barriers to prevent infections by generalist entomopathogens.

Therefore, our overall goal was to compare the infectivity of the fungal entomopathogen *Beauveria bassiana* strain GHA on two human-associated host species with disparate life histories: *C*. *lectularius* and *B. germanica*. By exposing bed bugs and cockroaches to conidia through different routes, including surface contact, topical treatment, ingestion, and injection, we sought to broadly identify and compare their barriers to fungal infection. These barriers include the exterior cuticle (surface contact, topical), the interior cuticle, digestive tract, and peritrophic membrane (ingestion), and the immunological barriers (injection). We also investigated whether suspending the conidia in oil or keeping them dry increased the infectivity of *B. bassiana*. The findings of this study provide important insights into the optimal routes of exposure to fungal entomopathogens for biocontrol of bed bugs and German cockroaches.

## 2. Materials and Methods

### 2.1. Insect Rearing and Bioassay Preparation

Colonies of *B. germanica* (Orlando normal strain) and *C. lectularius* (Harold Harlan strain) were maintained at 27 ± 1 °C, 12 L:12 D, and approximately 50% RH. These strains are reference insecticide-susceptible strains which were collected in 1947 and 1973, respectively. All experiments were conducted at 26 ± 1 °C, 12 L:12 D, and approximately 50% RH. The optimal growth temperature for *B. bassiana* GHA is 25 °C [14]. Cockroaches were supplied ad libitum with rodent chow (Purina Mills, St. Louis, MO, USA) and water in glass tubes that were stoppered with cotton. Newly emerged adult males were collected from a synchronously reared colony and maintained to an adult age of 20–30 days prior to use for all bioassays detailed below. Each cage (19 × 14 cm) had a paper egg carton as shelter.

Bed bug colonies were fed defibrinated rabbit blood in an artificial feeding system [15]. Briefly, a custom-made, water-jacketed glass condenser (Prism Research Glass, Raleigh, NC, USA) was used to contain and heat the blood. The internal chamber (blood reservoir) was surrounded by an outer chamber (circulating water reservoir) that was connected to a water circulator which transferred heated water through the condenser, which maintained the blood near human skin temperature (35 °C). Plant grafting tape (A.M. Leonard Horticultural Tool and Supply Co., Piqua, OH, USA) was stretched across the bottom of the feeder, and blood was pipetted into the internal chamber through an opening at the top. The bed bugs were housed in containers with folded paper for harborage, and the top was covered with plankton netting (BioQuip Products, Rancho Dominguez, CA, USA), through which all life stages could feed. Adult male bed bugs 14 days post emergence that had not been fed for 7–10 days were used for the experiments, with the exception of the injection experiments, which used bugs that had not been fed for 4 days.

### 2.2. Beauveria Bassiana GHA Preparation

Pure *B. bassiana* GHA (henceforth referred to as *B. bassiana*) conidia were provided by Dr. Nina Jenkins. The conidia were transferred to sterile containers and then suspended in sterile distilled water with 0.01% Tween-80 (99.5%, Sigma-Aldrich, St. Louis, MO, USA). Suspensions of different conidia concentrations were then created by serial dilution of the stock suspension. The conidia concentration of the suspensions was confirmed using an Improved Neubauer hemocytometer. Viability of the conidia was confirmed to be >98% by plating suspensions of known concentrations onto Petri dishes containing ~20 mL of sterile Sabouraud dextrose agar (Ward’s Science, Rochester, NY, USA). The conidia counts were confirmed for each of the experiments described below by plating an aliquot of the counted suspension on 20 mL of sterile Sabouraud dextrose agar in 90-mm plastic petri dishes.

### 2.3. Injection of B. bassiana Conidia

To assess the maximal efficacy of *B. bassiana* while bypassing the host cuticular barrier, we injected conidia suspended in 1X PBS (Apex, Genesee Scientific, San Diego, CA, USA) into the hemocoel of the insects. Ten cockroaches were used per replicate. Individuals were incapacitated by chilling (8 °C for 10 min) in a refrigerator before being placed dorsal side down onto a filter paper in a chilled 90-mm Petri dish and injecting 1 µL of a known concentration of conidia (4.4 × 10^2^ to 1.3 × 10^6^ conidia per insect) suspended in sterile 1X PBS using a 32-gauge beveled needle and 10-µL syringe (Hamilton, Reno, NV, USA). The injections were performed by hand, and entry was made between the 6th and 7th abdominal sternites away from the insect midline. After injection, the insects were transferred to a glass jar (60 × 100 mm) containing a pellet of rodent chow and a glass water tube stoppered with cotton. The control cockroaches were injected with either 1 µL of sterile 1X PBS or 1 µL of heat-killed (autoclaved) 1.3 × 10^6^ conidia.

For the bed bug injection assays, 10 adult males were used per replicate. The bugs were restrained dorsal side down on double-sided sticky tape that was attached to a glass microscope slide. We used an autoinjector (World Precision Instruments, Sarasota, FL, USA) with a needle drawn from a glass capillary (original capillary dimensions, 1.14 mm OD, 8.9 cm, No. 504949, World Precision Instruments) to inject the bed bugs with a 0.1-µL suspension of conidia in 1X PBS (4.4 × 10^2^ to 8.8 × 10^5^ conidia per insect). The control bed bugs were injected with either 0.1 µL of sterile 1X PBS or 0.1 µL of deactivated (autoclaved) 8.8 × 10^5^ conidia.

Three replicates per concentration (n = 30) were conducted with the cockroaches and bed bugs. After the injections, the insects were placed in a temperature-controlled room as described above, and mortality was scored every 24 h for 14 days. To confirm that mortality was caused by *B. bassiana*, mycosis was confirmed by placing the dead insects in a 14.8-mL cup with a wet ball of cotton. The cups containing dead insects were then placed in a separate environmental rearing chamber (12 L:12 D, 26 ± 1 °C, ~50% RH) and checked daily for sporulation of the fungi. The same timeline for mortality scoring and mycosis induction was used for all the experiments described below.

### 2.4. Ingestion of B. bassiana Conidia

To assess the infectivity of *B. bassiana* on cockroaches through the digestive tract, we adapted the method of Wada-Katsumata et al. [16]. Briefly, individual male cockroaches were placed into 90-mm glass Petri dishes and starved for 24 h. Conidia were then suspended in water + 0.01% Tween-80, and sucrose was added to create a 1 M sucrose + conidia solution. Then, 4 µL of the conidia + sucrose solution (4.4 × 10^6^ conidia) was pipetted onto a plastic microscope slide (22 × 22 mm), forming a stable liquid droplet. The microscope slide was transferred to a glass Petri dish housing a single cockroach, and the dish was covered to exclude light. The cockroaches were given 2 h to ingest the liquid droplet. The cockroaches that fully consumed the droplet (> 90%) were transferred to jars, and mortality was scored daily as described above. The control insects were fed 4 µL of a 1 M sucrose solution, and the positive controls were fed 80 µg of boric acid (2%) in 1 M sucrose + 0.01% Tween-80 to confirm that this was an effective method to deliver a toxicant to cockroaches.

The bed bugs were fed blood supplemented with conidia. Briefly, suspensions of conidia were prepared in sterile distilled water with 0.01% Tween-80. The conidia suspension (30 µL) was then added to 2.97 mL of rabbit blood (1% with conidia 0.001% Tween-80 solution, final concentration). Groups of 30 male bed bugs were then placed into feeding vials as described above and allowed to feed for 30 min. The dose of conidia ingested by each male (2.2 × 10^3^ to 4.4 × 10^6^ conidia per insect) was calculated based on the assumption that each male consumed ~4 µL of blood [15]. After feeding, the bed bugs were transferred to 100-mm glass Petri dishes (10 per dish) and placed in incubators as described above. The control bed bugs were fed blood with 1% sterile distilled water with 0.001% Tween-80 (final concentration when mixed in blood). Only the bed bugs that were fully engorged were analyzed. Each treatment group was replicated three times for both the cockroaches and bed bugs.

### 2.5. Topical Application of B. bassiana Conidia in Water or Oil

To compare the ability of *B. bassiana* to penetrate the exterior cuticle of the target insects when suspended in water or oil, we conducted topical application bioassays. Briefly, suspensions of sterile distilled water with 0.01% Tween-80 were prepared (8.8 × 10^4^ to 1.3 × 10^6^ conidia per insect). Suspensions of conidia in oil (from 8.8 × 10^4^ to 4.4 × 10^6^ conidia per insect) were prepared by concentrating or diluting a stock oil suspension of *B. bassiana* conidia (2.2 × 10^9^ conidia per ml, Aprehend^®^, Conidiotec, Centre Hall, PA, USA) with oil (Conidiotec). Next, the cockroaches and bed bugs were incapacitated using the refrigerator and placed dorsal side down onto a Petri dish before 1 µL of the conidia in water or oil was applied to the metathoracic area of each insect. The cockroaches were transferred to jars, and the bed bugs were transferred to Petri dishes. Each treatment group was replicated three times for both insect species. The control groups consisted of treatment with 1 µL of sterile distilled water with 0.01% Tween-80 or 1 µL of oil.

### 2.6. Surface Contact Bioassays with B. bassiana Conidia in Oil

To assess the ability of *B. bassiana* to infect cockroaches and bed bugs through contact with a treated surface, 90-mm filter papers (Whatman No. 1, Cytvia, Marlborough, MA, USA) were sprayed using an airbrush sprayer (Conidiotec) with either oil or *B. bassiana* conidia suspended in oil (8.8 × 10^4^ to 8.8 × 10^6^ conidia/cm^2^, while the label application rate of Aprehend^®^ for bed bugs is 4.4 × 10^6^ conidia/cm^2^). All suspensions were thoroughly agitated before use, and the airbrush was held horizontally to spray the filter papers. To ensure that the entire area of the filter papers was treated, two passes were made over them (one over each half) at a rate of 30 cm/sec. The airbrush was kept 10 cm from the filter papers. The treated filter papers were allowed to dry for 24 h and then placed in the lids of the 90-mm Petri dishes so that the base could form a tight seal and prevent escape. The insects were then cold-anesthetized and transferred to the Petri dishes. The cockroaches were provided with a water tube and rodent food that had the bottom covered so it did not contact the treated filter paper. The pellet was not tested for *B. bassiana* contamination, but no visible sporulation was observed from the rodent chow. The lids were then secured using labeling tape, and the Petri dishes were incubated at the conditions described above. The treatments and controls (oil) were replicated two and five times for the bed bugs and cockroaches, respectively.

### 2.7. Surface Contact with Liquid-Sprayed vs. Dry B. bassiana Conidia

Because the conidia that were suspended in water or oil were ineffective on the German cockroaches, we compared the infectivity of conidia suspended in water or oil with dry conidia that were deposited on a surface with both insects. We treated the 90-mm filter papers with conidia suspended in either water or oil with an airbrush sprayer as described above, resulting in 1.3 × 10^6^ conidia/cm^2^. After drying for 24 h, the treated filter papers were placed in 100 × 10 mm glass Petri dish lids so the insects only had access to the treated filter paper by using the base as the top (~0.5 cm was uncovered). Dry conidia (8.6 mg, equivalent to 1.3 × 10^6^ conidia/cm^2^) were placed on a 90-mm filter paper in a glass Petri dish and evenly spread using a fine paint brush. The insects were anesthetized and placed in the Petri dishes with food and water as described above. Each treatment group was replicated 3 times with 10 insects per replicate.

### 2.8. Statistical Analysis

The mortality of the insects exposed to different concentrations of conidia during each 14-day experiment was analyzed using Kaplan–Meier survival analysis in SPSS 27 (IBM, Armonk, NY, USA). Different treatments and control groups were then compared in a pairwise manner using a log-rank test. Insects that survived beyond the 14-day period were censored to indicate survival. Because survival analysis assumes equal probabilities of individuals leaving the study [17], we added one dead insect to all treatment groups on day 14 if no mortality occurred in the controls or other treatment groups. If the dummy replicate was not added when no mortality occurred, the analysis could not be conducted. When this occurred, the median survival times were still > 14 days for the control treatments. To determine the hazard ratios for different treatments, a single proportional hazard regression was conducted. The controls in each experiment were used as the baseline of comparison of hazard regression and statistical separation of different treatments. When possible, the mean survival times (MSTs), median survival times, and relative log hazard ratios were estimated.

## 3. Results

**Figure 1:**
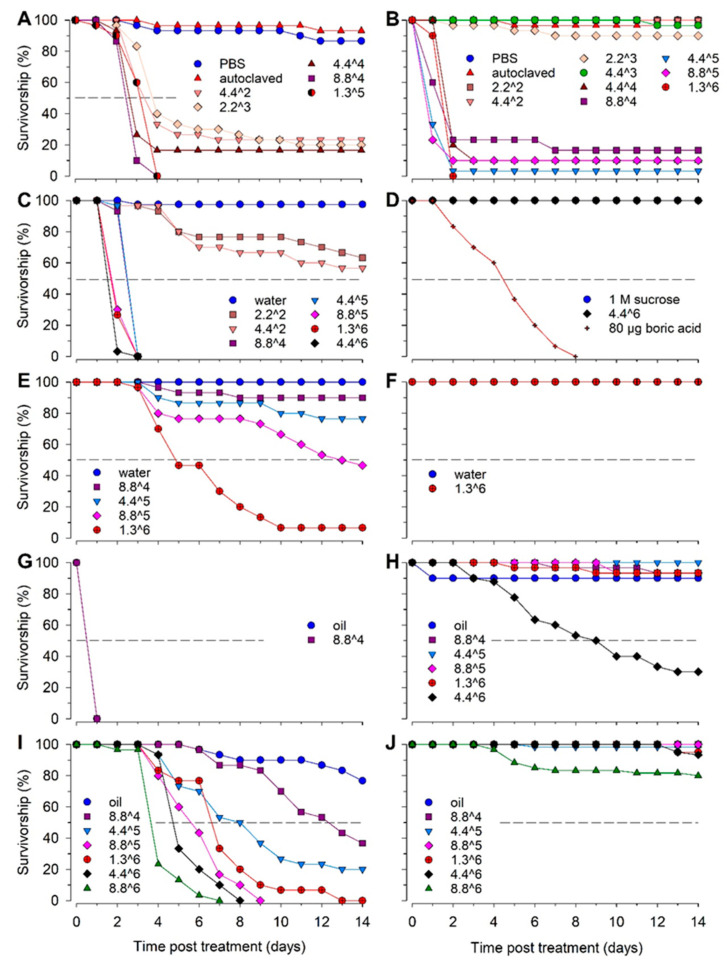
Mean proportional survival of bed bugs and German cockroaches exposed to *B. bassiana* GHA conidia via injection (**A**,**B**), feeding (**C**,**D**), topical application of conidia in water (**E**,**F**), topical application of conidia in oil (**G**,**H**), and contact with surfaces sprayed with conidia in oil (**I**,**J**). The exposure method dictated what control treatment the insects received. For example, the injection controls were injected with 1X PBS, whereas the topical application controls received water. More details on the different control types are included in the methods section. Data and lines overlap for both treatments in (**F**,**G**).

We assayed the infectivity of *B. bassiana* GHA on bed bugs and German cockroaches by delivering conidia to the insects using various means. We also assayed the effects of an oil formulation of conidia to determine if oil enhanced the infectivity of *B. bassiana*. Bed bug survivorship was significantly affected by *B. bassiana*, with all five routes of exposure (Table 1, Figure 1A,C,E,G,I) indicating their susceptibility to this entomopathogen. Conversely, different routes of exposure of German cockroaches to *B. bassiana* conidia resulted in different survivorship rates (Table 2, Figure 1B,D,F,H,J). Overall, however, the cockroaches were much less susceptible to *B. bassiana* compared to the bed bugs.

### 3.1. Injection of B. bassiana Conidia

We used two independent control injections—PBS and autoclaved conidia—to assess the effects of the injection on the bed bugs and cockroaches. In both species, injected PBS or autoclaved conidia resulted in minimal (<6.6% and 3.3%, respectively) mortality (Figure 1A,B), and cultures of the injected solutions produced no fungal growth. Injection of *B. bassiana* conidia into the hemocoel was an effective route of exposure for the bed bugs, with the two highest doses reaching 100% mortality in 2–3 days (overall model, chi-square = 90.5, d.f. = 5, *p* < 0.0001) (Table 1, Figure 1A). The mortality levels of these two high doses were significantly different from each other, due mainly to differences on day 2 (8.8 × 10^4^ conidia: MST = 2.9 ± 0.9 days, 1.3 × 10^5^ conidia: MST = 3.2 ± 1.4 days; chi-square = 6.6, d.f. = 1, *p* = 0.01). Even at lower doses (4.4 × 10^2^ to 4.4 × 10^4^ conidia per insect), we achieved high mortality (76.6–83.3%). It is notable, however, that the pairwise comparisons showed no significant differences among these three doses (chi-square = 0.8–3.6, d.f. = 1, *p* > 0.05, MST = 5.0–6.4 days). The overall high infectivity of *B. bassiana* in bed bugs after bypassing the cuticular barrier suggests that they are highly susceptible to *B. bassiana*.

Although injection was also the most effective route of exposure for German cockroaches, *B. bassiana* was much less infective against cockroaches than bed bugs (Table 1, Figure 1B). Low doses of conidia (<4.4 × 10^4^) caused low mortality, but at higher doses, the cockroaches experienced high mortality, which mostly occurred 1–2 days after injection. Thus, there was a clear separation of survivorship curves above and below 4.4 × 10^4^ conidia (overall model, chi-square = 170.4, d.f. = 5, *p* < 0.0001). There was a generally broad statistical overlap among the high-dose treatments, but two treatments were significantly different (4.4 × 10^4^ conidia: MST = 4.2 ± 1.0 days, 4.4 × 10^5^ conidia: MST = 2.2 ± 0.5 days; chi-square = 59.98, d.f. = 1, *p* < 0.001). Only one of the doses (1.3 × 10^6^ conidia) caused 100% mortality.

### 3.2. Ingestion of B. bassiana Conidia

The *B. bassiana* conidia were effective when fed to bed bugs (overall model, chi-square = 145.3, d.f. = 5, *p* < 0.001), with high doses causing 100% mortality by day 3 (chi-square = 5.4–63.3, d.f. = 1, *p* < 0.05 for all pairwise comparisons) (Figure 1C). Similar to the injection experiments with cockroaches, we observed a threshold effect, where low doses (≤4.4 × 10^2^) resulted in > 50% survivorship estimates after 14 days, whereas higher doses were much more effective at killing the bed bugs (MSTs: 8.8 × 10^4^ conidia = 2.93 ± 0.05 days, 1.3 × 10^6^ conidia = 2.2 ± 0.08 days; 4.4 × 10^6^ conidia = 2.03 ± 0.03 days; chi-square = 50.4, d.f. = 1, *p* < 0.001).

We validated our feeding method for the cockroaches by incorporating 80 µg of boric acid into the sugar solution. All cockroaches died by day 8, as expected (Figure 1D). However, a high dose of 4.4 × 10^6^ conidia per cockroach failed to kill any cockroaches in the feeding assays. These results indicate that *B. bassiana* conidia are ineffective at infecting cockroaches through the digestive tract.

### 3.3. Topical Application of B. bassiana Conidia

The topically applied conidia suspended in water were less effective on the bed bugs compared with the injected or ingested conidia. Nonetheless, significant differences were evident among the doses (overall model, chi-square = 101.6, d.f. = 4, *p* < 0.001). Topical application of ≤ 8.8 × 10^5^ conidia caused 10–53.3% mortality (MSTs 11.2–13.2 days), whereas 1.3 × 10^6^ conidia, the highest dose we tested, caused 93.3 ± 3.3% mortality 14 days after treatment (MST = 6.6 ± 0.56 days, chi-square = 59.98, d.f. = 1, *p* < 0.001) (Figure 1E). Bed bug mortality began on days 3 and 4 and slowly increased until day 10.

Topical application of the highest *B. bassiana* dose of 1.3 × 10^6^ conidia suspended in water caused no mortality in the cockroaches (Figure 1F). These results demonstrate that the *B. germanica* cuticle constitutes an effective barrier to *B. bassiana* conidia or that the wetting of *B. bassiana* conidia significantly impacts its ability to penetrate past the cuticle and enter the hemocoel.

Topical application of conidia in oil appeared to be effective against the bed bugs, with all tested doses causing 100% mortality in 1 day (overall model, chi-square = 115.4, d.f. = 6, *p* < 0.001) (Figure 1G). However, the oil itself was toxic to bed bugs, also causing 100% mortality in 1 day. Therefore, we could not uncouple the effects of *B. bassiana* conidia from those of the oil carrier.

In contrast to the bed bugs, the oil by itself caused only 10% mortality in the male German cockroaches on day 1, and 90% survived to day 14 (Figure 1H). Therefore, we could assess the effects of *B. bassiana* conidia on the cockroaches. Topical treatment with ≤1.3 × 10^6^ conidia had minimal effects which were not significantly different from those of oil alone (chi-square = 0.17, d.f. = 6, *p* = 0.68). However, topical application of 4.4 × 10^6^ conidia in oil caused significant mortality (overall model, chi-square = 115.4, d.f. = 6, *p* < 0.001), with 70% killed within 14 days (MST = 9.4 ± 0.8 days, chi-square = 20.16–32.85, *p* < 0.001 for all pairwise comparisons with 4.4 × 10^6^ conidia). Thus, it appears that oil slightly enhanced the efficacy of *B. bassiana* against German cockroaches.

### 3.4. Surface Contact with B. bassiana Conidia

We applied conidia suspended in oil to filter papers, and 24 h later, bed bugs or cockroaches were placed on the filter papers. All the bed bugs survived for 14 days on the water-treated control filter papers, and 23.3% experienced mortality on the oil-treated control filter papers (Figure 1I). Contact with conidia applied in oil caused mortality in the bed bugs in a dose-dependent manner, starting 3–4 days after the start of the experiment (overall model, chi-square = 206.5, d.f. = 7, *p* < 0.001). Concentrations ≥8.8 × 10^6^ conidia/cm^2^ killed 100% of the bed bugs during the 14-day exposure period, with significantly lower MST estimates at higher concentrations (4.4 ± 0.2 days at 8.8 × 10^6^ conidia/cm^2^, 6.5 ± 0.4 days at 8.8 × 10^5^ conidia/cm^2^; chi-square = 21.37, d.f. = 1, *p* < 0.001). These results demonstrate that the *B. bassiana* conidia suspended in oil and sprayed onto a filter paper surface were effective against bed bugs by contact.

In contrast to the bed bugs, the German cockroaches (Figure 1J) were resilient to the oil formulation of *B. bassiana*. The oil alone, when applied to the filter papers, caused no mortality in the cockroaches, and only 20% mortality occurred after 14 days of continuous contact with the highest concentration of 8.8 × 10^6^ conidia/cm^2^ (overall model, chi-square = 34.7, d.f. = 7, *p* < 0.001). The mean survival time at this concentration was 12.5 ± 0.4 days, significantly lower than those at all other concentrations (chi-square = 4.6–9.8, *p* < 0.05 for all pairwise comparisons).

### 3.5. Comparison of Infectivity of Sprayed vs. Dry B. bassiana Conidia

**Figure 2:**
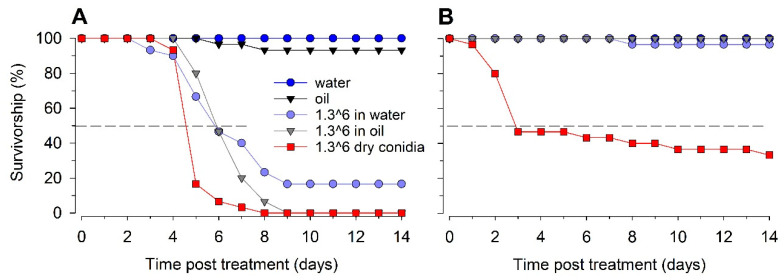
Mean proportional survival of bed bugs (**A**) and German cockroaches (**B**) in contact with filter papers treated with *B. bassiana* GHA conidia that were deposited on filter papers as dry conidia or suspended in water + 0.01% Tween-80 or oil and sprayed on filter papers. Control filter papers were treated with water or oil alone.

We found significant differences among the three treatment groups (dry conidia applied directly to the filter paper surface and conidia suspended in water or oil and sprayed onto the filter paper) and two control groups (water or oil sprayed on filter papers) (overall model, chi-square = 153.53, d.f. = 4, *p* < 0.001). The bed bugs exposed to dry conidia experienced a mean survival time of 5.5 ± 0.3 days, representing significantly faster mortality than that in the bed bugs exposed to surfaces sprayed with conidia suspended in water or oil (chi-square = 10.6–70.7, *p* < 0.001 for all pairwise comparisons) (Table 3, Figure 2A). Equivalent high rates of conidia suspended in oil and in water (1.3 × 10^6^ conidia/cm^2^) resulted in similar survivorship (chi-square = 1.2, d.f. = 1, *p* = 0.27), with mean survival times of 6.8 ± 0.3 days and 7.4 ± 0.6 days, respectively, and 100% mortality by day 14. The two control treatments had similar survivorship (chi-square = 1.0, d.f. = 1, *p* = 0.29), with no mortality in the water control group and 6.6% mortality in the oil control group.

We found significant differences in the survivorship of German cockroaches in the three treatments and two control groups (overall model, chi-square = 99.5, d.f. = 4, *p* < 0.001) (Figure 2B). A comparison of equivalent rates of conidia (1.3 × 10^6^ conidia/cm^2^) suspended in water or oil demonstrated that both treatments were ineffective against the cockroaches, with only 3.3% mortality in cockroaches exposed to conidia sprayed in water and 0% mortality in cockroaches exposed to conidia sprayed in oil, consistent with previous findings (Figure 1J). These two treatment groups were similar to each other as well as the two control groups, which experienced no mortality (chi-square, *p* > 0.55 for other comparisons). In contrast, the cockroaches exposed to dry conidia reached 66% mortality by the end of the 14-day experiment, with a mean survival time of 7.6 ± 0.9 days, which was significantly different from those of the other treatments (pairwise chi-square = 29.9, *p* < 0.001). These results demonstrate striking differences in the infectivity of *B. bassiana* conidia applied in dry form or as suspensions in water or oil. The conidia were much less infective when sprayed in a liquid suspension than when applied in dust form. Although this loss of effectiveness was evident against both bed bugs and German cockroaches, it was particularly severe with the cockroaches.

We confirmed mycosis in 100% of the cadavers of insects exposed to *B. bassiana* in all the above-mentioned bioassays. There was no evidence of sporulation under high humidity conditions in the bed bugs that were killed by the oil alone.

## 4. Discussion

The infectivity of host insects by fungal entomopathogens depends on a variety of factors, including environmental conditions, characteristics related to the biology of the host, and features of the pathogen itself. In this study, we compared the infectivity of the fungal entomopathogen *Beauveria bassiana* GHA, which has a broad host range, on two sympatric synanthropic pests with divergent life histories: the bed bug (*Cimex lectularius*) and the German cockroach (*Blattella germanica*). By using different modes of exposure, we sought to determine the extent to which various barriers prevent fungal entomopathogen infection that could potentially be overcome with further studies. Although some modes of exposure were more effective than others, we found that, overall, the bed bugs were susceptible to *B. bassiana* through all the tested routes of exposure. These findings are consistent with previous research using contact assays with the same *B. bassiana* strain [18,19,20]. On the other hand, the German cockroaches were largely unaffected by *B. bassiana*, except when conidia were introduced directly into the hemocoel by injection or when exposed to dry aerial conidia that were not sprayed in a liquid suspension. Below, we consider different factors that might contribute to the susceptibility of bed bugs and resilience of cockroaches to *B. bassiana*.

### 4.1. Evolutionary History

Bed bugs may not have evolved strong defense mechanisms against fungal entomopathogens because of their unique evolutionary history and current ecology. Most cimicids are associated with and primarily feed upon bats and birds, but *C. lectularius* and *Cimex hemipterus* have adapted to feed on human blood and infest residential settings [21,22]. Human-associated bed bugs evolved from ancestral cimicids that specialized in bats. The switch to using humans as hosts likely occurred in caves, where bugs descended from bat roosts to feed upon the blood of cave-dwelling humans when their preferred hosts were unavailable [21,22]. Although soil and cave environments have abundant microbiota that are relatively shielded from radiation and the environment [23,24], bed bugs often harbor near their hosts, which could have resulted in minimal contact with soil-dwelling entomopathogens in caves or other microorganisms in human-built environments throughout their evolutionary history. Moreover, bed bugs feed on blood which is essentially sterile. Bed bugs therefore might not have experienced strong selection pressures to evolve adaptations to prevent fungal entomopathogen infection.

Conversely, cockroaches occupy a wide variety of habitats and feed on an array of resources, such as decaying organic material in leaf litter, leaves, algae and other epiphylls on leaf surfaces, and decaying wood [25,26]. Most cockroaches are omnivores, using their chewing mouthparts to sample and consume a variety of microbe-rich foods from decaying material and soil [13,25]. Indeed, many of these microbes become members of the cockroach gut microbiota [27], and the gut microbiome of the German cockroach is considerably more diverse and abundant than that of bed bugs [27,28,29]. Therefore, it is likely that the German cockroach evolved a variety of physical barriers, behaviors, and physiological mechanisms that prevent infection by various entomopathogens.

### 4.2. Penetration of the Cuticle

The insect cuticle represents the first barrier against environmental stressors, desiccation, and entomopathogen infection [30,31]. Features of the cuticle, such as its thickness, degree of melanization and sclerotization, and the lipid layer on the epicuticle, also influence its effectiveness at preventing entomopathogen penetration [32]. Cuticular hydrocarbons can either stimulate or inhibit the growth of conidia [33,34]. For example, in vitro digestion assays, where cockroach wing pads, oothecae, and thoraxes were submerged in *Conidiobolus coronatus* Batko (Costantin)-containing solutions, revealed that some free fatty acids were inhibitory, whereas others were susceptible to fungal degradation [34]. Gutierrez et al. [31] found that oleic acid, palmitic acid, and linoleic acid were predominant fatty acids in the German cockroach cuticle. In vitro assays revealed that palmitic and linoleic acid had anti-fungal activity against the phytopathogenic fungi *Alternaria solani*, *Fusarium oxysporum*, and *Colletotrichum lagenarium* [35]. These limited observations suggest that the thick cuticular lipid layer, which likely evolved to prevent water loss [36,37], also protects the German cockroach from entomopathogen invasion. Overall, it appears that both the quantity and the quality of hydrocarbons and fatty acids (chain length, methyl branches, and degree of unsaturation) may affect conidia viability and penetration of the cuticle.

Moreover, whereas the bed bug cuticle is approximately 10 µm thick [38], the thickness of the German cockroach cuticle is around 16 µm [39]. The German cockroach cuticle is highly melanized, which also offers protection against desiccation [40]. The overall effectiveness of the cuticle as a barrier is highlighted by the high susceptibility of the German cockroach to injected conidia at ≥8.8 × 10^4^ and its complete resilience to 1.3 × 10^6^ conidia applied directly to the cuticle. In contrast, the bed bug cuticle must remain pliable to accommodate vast expansion of the abdomen with large blood meals, and because it has an ample supply of water in each blood meal, it appears to invest much less in the production of cuticular lipids. The collateral effect may be the susceptibility of bed bugs to cuticular penetration by fungal pathogens when exposed through different routes, as was observed in this study.

### 4.3. Allelochemicals and Grooming Behavior

Bed bugs produce short-chain aldehydes as alarm and aggregation pheromones [41]. Two aldehydes, (*E*)-2-hexenal and (*E*)-2-octenal, suppress *M. anisolpliae* growth in vitro, and (*E*)-2-hexenal at >200 µg was also antagonistic to *B. bassiana* [42]. However, this is much more (*E*)-2-hexenal than bed bugs normally produce [41], so it remains to be determined whether bed bug aldehydes can suppress conidial growth at biologically relevant concentrations.

Bed bugs and cockroaches evolved divergent self-grooming strategies that could influence entomopathogen infectivity. During grooming, hemipterans concentrate external particles on the tarsi and tibia and then dislodge them by rubbing against a surface [43]. This grooming strategy could transfer conidia to the highly articulated terminal segments of the legs, where they could then penetrate the cuticle more readily. Further research is needed to understand whether grooming aids bed bugs in removing fungal conidia or facilitates conidial penetration of the cuticle.

In contrast, German cockroaches groom extensively to improve chemoreception and courtship success, and this process also removes environmental contaminants and microorganisms from the exterior of the insect [44,45,46]. Grooming concentrates contaminants from the cuticle to an appendage—usually a foreleg—and the concentrated material is then ingested [43,46,47]. Fungal conidia would then enter the cockroach’s digestive tract and be inactivated by a combination of anti-microbial compounds produced by the cockroach and commensal microorganisms [48,49], the gut pH, and digestive enzymes [50]. Therefore, the grooming behavior in bed bugs may deliver conidia to favorable cuticular sites, whereas in the cockroach, grooming likely results in conidia being inactivated.

In support of this hypothesis, *Cryptocercus*, a subsocial cockroach whose ancestors may have given rise to termites, lives inside and feeds upon wood and has antifungal compounds in salivary secretions and on the cuticle that are used in self- and allogrooming of nestmates [51]. The *Cryptocercus* hindgut also has abundant β-1,3-glucanase activity, and their fecal pellets have antifungal properties [52]. Both of these adaptations in *Cryptocercus* help prevent pathogen outbreaks in conditions that are favorable to fungal growth. Termites also possess similar adaptations. In simulated colony set-ups of the subterranean *Reticulitermes flavipes*, they were able to inhibit germination of *Metarhizium anisopliae* conidia [53]. Termites are also highly efficient at grooming and removing fungal conidia [54]. The conidia then enter the termite gut, a fungistatic environment with multiple antifungal compounds that prevent the germination of conidia despite contact with the cuticle [55]. The gut microbiome of *Zootermopsis angusticollis* produces multiple β-1,3 glucanases, which are antifungal [56]. The fecal pellets of *Zootermopsis angusticollis*, which are embedded in the nest structure, also have antifungal properties and prevent fungal outbreaks [57]. Because of the phylogenetic proximity of termites to cockroaches, *Blattella germanica* may share some of these antifungal adaptations [48,49].

### 4.4. Penetration through the Digestive Tract Tissue

Bed bugs are highly susceptible to ingested insecticides [15,58] and bacterial pathogens [59]. Our present findings show that bed bugs are also susceptible to *B. bassiana* through feeding. Notably, hemipterans lack a defined peritrophic membrane [60,61], a chitinous envelope that forms around a food bolus as it enters the insect midgut and protects the basal lamina of the midgut (which lacks a cuticle) from abrasion and infection while feeding [62]. Hemipterans may have lost the ability to produce a defined peritrophic membrane because they feed primarily on sterile fluids, namely either plant sap or host blood [60,61]. Humoral immune responses related to the NK-kB pathway play a role in bed bug immunity to ingested entomopathogenic bacteria, reducing the infectivity of *Bacillus thuringiensis israelensis* and *Pseudomonas entomophilia* [63]. However, the barriers that bed bugs have against ingested *Beauveria* conidia and other entomopathogens are less able to prevent infection.

The German cockroach, on the other hand, produces a well-organized peritrophic membrane whose components are upregulated by food intake [64]. It is also effective at preventing pathogenic microorganisms from interacting with the gut microvilli [32]. Therefore, as the German cockroach ingested conidia, its peritrophic membrane shielded the midgut from the conidia, and the conidia were ineffective even at the highest dose tested (4.4 × 10^6^ conidia).

### 4.5. Hemolymph and Immune Defenses

The final layer of defense against fungal conidia is the hemolymph and the associated immune system. The insect hemolymph is a nutrient-rich environment for fungal conidia to germinate, but it also contains hemocytes capable of encapsulation and phagocytosis of pathogens and antimicrobial peptides [65]. Bed bugs have a well-developed immune system, possibly related to traumatic insemination, which wounds the cuticle and could facilitate the entry of microorganisms [66]. Female bed bugs have a specialized mesospermalage that is adapted to neutralizing microorganisms that enter the hemocoel in this area [67]. Although the immune system is primed by feeding in anticipation of mating damage and pathogen exposure [67,68], the immune response may be localized in the spermalage area and not offer broad protection throughout the hemocoel. Such regionalization would be consistent with the general susceptibility of bed bugs to bacterial entomopathogens when pierced by a pathogen-contaminated needle [59] as well as injections of *B. bassiana* conidia, as in the present study. The bed bug genome includes genes with putative immune functions (as members of the Toll, Imd and Jak/STAT pathways), but the diversity of immune genes is sparse compared with other insect genomes. Both recognition proteins and antimicrobial peptides are particularly under-represented in bed bugs [69]. The overall susceptibility of bed bugs to *B. bassiana* is further supported by our observation that similar doses were nearly equally effective by injection and ingestion. For example, a minimal dose of 4.4 × 10^2^ conidia was slightly more effective by injection, but a dose of 8.8 × 10^4^ was equally effective by injection and ingestion, causing 100% mortality by day 4.

Injection into the hemocoel was the only route of delivery of suspended *B. bassiana* conidia that was effective against the German cockroaches. We observed a sharply delineated threshold, where doses <4.4 × 10^4^ conidia were ineffective while higher doses killed >85% of the treated cockroaches. These observations suggest that the higher doses of conidia can overcome the immune system of the cockroach. The hemolymph of *B. germanica* contains a variety of immunocytes and other hemocytes [70]. For example, plasmatocytes and granulocytes, which can encapsulate or phagocytize conidia, respectively, increase in number in response to infection with *Aspergillus* [71]. Several gene families in the cockroach genome, with functions in microbial defense and immune response, have expanded, likely in the ancestral Blattodea lineage, unlike in the bed bug genome. Among the 10 clusters of 195 genes encoding putative defense proteins, the hemolymph lipopolysaccharide (LPS)-binding proteins (86 genes) that facilitate phagocytosis by hemocytes represent a massive gene expansion [72]. Recent work on *B. germanica* revealed 39 AMP genes, which had not initially been annotated and likely play an important role in microbial immunity [73]. Other notable expansions include 10 drosomycin copies and 8 glucosylceramidases that serve as hemolymph antifungal peptides. These expansions in gene families with functions related to microbial defense and immune response presumably evolved under selection pressure of the septic conditions in which *B. germanica* thrives.

### 4.6. Fungal Entomopathogens in the Indoor Environment

Sparse research has been conducted on the effectiveness of other fungal entomopathogens against *C. lectularius*, but *B. bassiana* has been consistently effective against bed bugs. Experiments with *B. germanica*, however, have yielded mixed results, with both high and low efficacy of different *B. bassiana* strains. We suspect that such disparate results may be due to differences in methodology, the deployment of conidia, and the virulence of the fungal strains. For example, unlike our findings, other researchers found *B. bassiana* to be effective against *B. germanica* in feeding experiments [31,74]. These differences could be due to unique ingredients in formulated bait products, longer exposure of cockroaches to the treated food (single feeding in our assays vs. 3 days of feeding in others), and the volume of conidia applied to the food (4 µL vs. 1 mL). Nevertheless, there is general agreement that topical application and direct sprays of *B. bassiana* are only marginally effective against *B. germanica* [48,75], and this is consistent with our findings that the *B. bassiana* strain GHA was relatively ineffective against *B. germanica* by all practical routes of exposure. On the other hand, *M. anisopliae* has been proven to be more effective against the German cockroach by both contact and ingestion [76,77]. *M. anisopliae* may be more effective against cockroaches, since they use only one type of insecticidal secondary metabolite, destruxin, to induce host mortality [78,79], whereas *B. bassiana* relies upon the cumulative effect of secondary metabolites [80,81].

These disparities led us to consider how conidia are formulated and delivered to the insects. The use of water or oil to suspend conidia, as well as surfactants such as Triton X-100 or Tween-80, can affect the adhesive properties of conidia and reduce the infectivity of entomopathogens [33,82]. Thus, “dry aerial conidia” resulted in greater mortality of *Culicoides nubeculosus* (Meigen) and *Hylobius abietis* (Linnaeus) than “wet conidia” that were suspended in water with a surfactant [81,82]. Our direct comparison of equivalent doses of *B. bassiana* conidia suspended in water, oil, or in dust form revealed that only the dry conidia caused mortality in *B. germanica*. These findings are consistent with previous work, showing that dry aerial conidia of *M. anisopliae* caused high mortality in *B. germanica* [76]. In contrast, all three treatments with *B. bassiana* conidia caused high mortality in bed bugs, although the dry conidia were most effective. Overall, the results with cockroaches and bed bugs suggest that *B. bassiana* conidia are somehow affected by being suspended in water or oil, making it more difficult for conidia to be dislodged from the surface, adhere to the cuticle, or penetrate the cuticle. Aerial conidia have a negative electrostatic charge [80] for initial adhesion to the insect cuticle [33]. Therefore, when exposed to dry aerial conidia, areas of different charges on the cockroach [83] and bed bug cuticle [84] allow for infective spores to be more readily picked up by the insects compared with conidia that are suspended in oil or water. Further research is needed to determine the extent to which formulation and surfactants influence infectivity and insect mortality when *B. germanica* and *C. lectularius* are exposed to dry and previously wet conidia.

Aside from selecting more infective strains, two broad strategies to improve the efficacy of entomopathogens include (1) increasing penetration of the cuticle and gut and (2) protecting conidia from the harsh environments where the host insect lives. Petroleum-based oils appear to do both by increasing infectivity at low humidity [85], protecting fungal conidia from environmental damage, and helping them spread on, adhere to, and penetrate the insect cuticle [86]. Topical application of *B. bassiana* in oil improved the efficacy of conidia against *B. germanica*, but we could not attain more than 70% mortality with a high dose of 4.4 × 10^6^ conidia in oil. In bed bugs, however, the oil by itself caused 100% mortality within a day, precluding any inferences about its role in facilitating the infectivity of conidia. Oils have suffocating activity in sufficient volumes [86], but we applied only 1 µL of oil away from any of the spiracles. We also repeated this procedure with acetone and found that it was not as bioactive as the oil.

Boric acid also synergizes the infectivity of entomopathogens when applied to or fed to *B. germanica* [74] by disrupting the peritrophic membrane, midgut cells, and the gut microbiota [87]. Talc powder, composed of inorganic insoluble microparticles (of magnesium silicate), also enhanced the efficacy of *M. anisopliae* against *B. germanica*, but the mechanism remains unknown [77]. Insecticides can also enhance entomopathogenic fungi against *B. germanica* [88] and *C. lectularius* [89], likely by causing stress and interfering with immune pathways [90]. Interestingly, adipokinetic hormone, which mobilizes energy substrates in response to stress [91], also promotes infections by *Isaria fumosorosea* in the cockroach *Periplaneta americana* [92]. It is clear from the findings presented here and related works that the effective use of fungal entomopathogens will require substantial investment in the discovery of infective fungi, synergistic compounds, and deployment strategies that enhance the infectivity of entomopathogens through contact and feeding.

## 5. Conclusions

Bed bugs are generally susceptible to *B*. *bassiana* GHA conidia by exposure via surface contact, topical application, feeding, and injection. The high mortality of bed bugs in the feeding experiments was likely due to the lack of a well-defined peritrophic membrane. The German cockroach was exceptionally resilient to infection by *B*. *bassiana* GHA, likely due to adaptive mechanisms that prevent infections, such as physical and chemical cuticular barriers, self-grooming behavior, high turnover of its peritrophic membrane, and several expanded pathways of antimicrobial and immune response proteins. Oil formulations enhanced the infectivity of *B*. *bassiana* GHA conidia in *B. germanica*, but the oil itself was toxic to bed bugs, precluding an analysis of the effects of conidia in oil. Finally, dry aerial conidia caused greater mortality in both bed bugs and cockroaches than conidia suspended in either water or oil. These findings confirm that fungal entomopathogens should be considered for bed bug control and highlight the importance of discovering strategies that enhance the infectivity of fungal entomopathogens against *B. germanica*. 

## Figures and Tables

**Table 1:** Kaplan–Meier estimates of mean survival time ± standard error (SE) for adult male Harold Harlan bed bugs exposed to *B. bassiana* GHA conidia through different routes. The dose of conidia delivered per insect is reported for all routes except for the contact experiments, where the values are reported in conidia per cm^2^. Significantly different treatments, as determined by a log-rank test, are indicated by different letters in the grouping column. Relative log hazard ratios that could not be calculated are denoted by a dash. Reference treatments used as the baseline for comparisons are indicated by Ref.

Exposure Route	Treatment	Group	Mortality (%)	Mean Survival Time ± SE (Days)	Median Survival Time (Days)	Relative Log Hazard Ratio ± SE
Injection	PBS	A	16.6	13 ± 0.6	≥14	Ref
	4.4 × 10^2^	B	76.6	6.3 ± 0.9	4	6.7 ± 0.5
	8.8 × 10^3^	B	80.0	6.4 ± 0.8	4	6.5 ± 0.5
	4.4 × 10^4^	B	83.3	5.0 ± 0.8	4	10.2 ± 0.5
	8.8 × 10^4^	C	100	2.9 ± 0.1	3	19.4 ± 0.5
	1.3 × 10^5^	D	100	3.3 ± 0.1	3	13.5 ± 0.5
Feeding	Water	A	3.3	13.6 ± 0.4	≥14	Ref
	8.8 × 10^4^	B	100	2.9 ± 0.04	3	-
	4.4 × 10^5^	B	100	2.9 ± 0.03	3	-
	8.8 × 10^5^	C	100	2.3 ± 0.08	2	-
	1.3 × 10^6^	C	100	2.2 ± 0.08	2	-
	4.4 × 10^6^	D	100	2 ± 0.03	2	-
Topical, water	Water	A	0	≥14	≥14	Ref
	8.8 × 10^4^	AB	10.0	13.2 ± 0.5	≥14	1.5 ± 1.1
	4.4 × 10^5^	BC	23.3	12.3 ± 0.7	≥14	2.2 ± 1.0
	8.8 × 10^5^	CD	53.3	11.2 ± 0.7	14	3.0 ± 1.0
	1.3 × 10^6^	E	93.3	6.7 ± 0.6	5	4.5 ± 1.0
Topical, oil	Water	A	0	13.7 ± 0.2	≥14	Ref
	Oil	B	100	1	1	2.7 ± 0.8
	8.8 × 10^4^	B	100	1	1	2.8 ± 0.8
	4.4 × 10^5^	B	100	1	1	2.8 ± 0.8
	8.8 × 10^5^	B	100	1	1	2.8 ± 0.8
	1.3 × 10^6^	B	100	1	1	2.8 ± 0.8
	4.4 × 10^6^	B	100	1	1	2.8 ± 0.8
Contact	Water	A	0	≥ 14	≥14	Ref
	Oil	A	23.3	13.5 ± 0.4	≥14	0.8 ± 1.2
	8.8 × 10^4^	B	63.3	11 ± 0.5	11	3.3 ± 1.0
	4.4 × 10^5^	C	80.0	7.3 ± 0.5	7	4.7 ± 1.1
	8.8 × 10^5^	C	100	6.5 ± 0.3	7	5.2 ± 1.1
	1.3 × 10^6^	C	100	6.6 ± 0.4	5	5.2 ± 1.1
	4.4 × 10^6^	D	100	5.4 ± 0.2	4	5.8 ± 1.1
	8.8 × 10^6^	E	100	4.4 ± 0.2	3	6.7 ± 1.1

**Table 2:** Kaplan–Meier estimates of mean survival time ± standard error (SE, not reported when mortality did not reach 50%) for adult male Orlando Normal German cockroaches exposed to *B. bassiana* GHA conidia through different routes. The dose of conidia delivered per insect is reported for all routes except for the contact experiments, where the values are reported in conidia per cm^2^. Significantly different treatments, as determined by a log-rank test, are indicated by different letters in the grouping column. Relative log hazard ratios that could not be calculated are denoted by a dash. Reference treatments used as the baseline for comparisons are indicated by Ref.

Exposure Method	Treatment	Group	Mortality (%)	Mean Survival Time ± SE (Days)	Median Survival Time (Days)	Relative Log Hazard Ratio ± SE
Injection	PBS	A	0	≥14	≥14	Ref
	4.4 × 10^2^	A	0	≥14	≥14	0 ± 1.4
	2.2 × 10^3^	A	10.0	12.9 ± 0.5	≥14	1.4 ± 1.1
	4.4 × 10^4^	B	83.3	4.2 ± 1	2	4.0 ± 1.0
	4.4 × 10^5^	C	90.0	2.2 ± 0.5	1	4.4 ± 1.0
	1.3 × 10^6^	B	100	2.3 ± 0.4	2	4.0 ± 1.0
Feeding	Water	-	0	-	≥14	Ref
	1.3 × 10^6^	-	0	-	≥14	-
Topical, water	Water	-	0	-	≥14	Ref
	1.3 × 10^6^	-	0	-	≥14	-
Topical, oil	Water	A	3.3	13.2 ± 0.7	≥14	Ref
	Oil	A	10.0	12.7 ± 0.8	≥14	-
	8.8 × 10^4^	A	6.7	13.5 ± 0.5	≥14	-
	4.4 × 10^5^	A	0	13.7 ± 0.2	≥14	-
	8.8 × 10^5^	A	6.7	13.8 ± 0.3	≥14	-
	1.3 × 10^6^	A	6.7	13.7 ± 0.3	≥14	-
	4.4 × 10^6^	B	70.0	9.4 ±0.8	9	2.5 ± 0.6
Contact	Oil	A	0	13.7 ± 0.4	≥14	Ref
	8.8 × 10^4^	A	1.7	13.9 ± 0.1	≥14	-
	4.4 × 10^5^	A	3.3	≥14	≥14	-
	8.8 × 10^5^	A	5.0	≥14	≥14	-
	1.3 × 10^6^	A	3.3	≥14	≥14	-
	4.4 × 10^6^	A	6.7	13.9 ± 0.1	≥14	-
	8.8 × 10^6^	B	20.0	12.5 ± 0.4	≥14	2.3 ± 1.0

**Table 3:** Kaplan–Meier estimates of mean survival time ± standard error (SE, not reported when mortality did not reach 50%) for adult male Orlando Normal German cockroaches and Harold Harlan bed bugs exposed to *B. bassiana* GHA conidia deposited on filter papers as dry conidia, in water + 0.01% Tween-80, or in an oil solution. The values are reported in conidia per cm^2^. Significantly different treatments, as determined by a log-rank test, are indicated by different letters in the grouping column. Relative log hazard ratios that could not be calculated are denoted by a dash. Reference treatments used as the baseline for comparisons are indicated by Ref.

Exposure Method	Treatment	Group	Mortality (%)	Mean Survival Time ± SE (Days)	Median Survival Time (Days)	Relative Log Hazard Ratio ± SE
Cockroaches	Water	A	0	≥14	≥14	Ref
	Oil	A	0	≥14	≥14	-
	1.3 × 10^6^ (water)	A	3.3	13.8 ± 0.3	≥14	0.7
	1.3 × 10^6^ (oil)	A	0	≥14	≥14	-
	1.3 × 10^6^ (dust)	B	66.6	7.7 ± 1	4	3.5 ± 1.0
Bed bugs	Water	A	0	≥14	≥14	Ref
	Oil	A	6.6	13.6 ± 0.4	≥14	1.1 ± 1.1
	1.3 × 10^6^ (water)	B	83.3	7.4 ± 0.6	6	4.0 ± 1.0
	1.3 × 10^6^ (oil)	C	100	6.7 ± 0.3	6	4.4 ± 1.0
	1.3 × 10^6^ (dust)	D	100	5.5 ± 0.3	5	5.0 ± 1.0

## Data Availability

Data will be made available upon request.
